# Coexistence of IgG4-Related Disease and Reactive Granuloma to Paraffin Plombage

**DOI:** 10.7759/cureus.40620

**Published:** 2023-06-19

**Authors:** Atsushi Isoda, Yukiko Sairenji, Masahiro Mihara, Hirono Iriuchishima, Akio Saito

**Affiliations:** 1 Department of Hematology, Iryohojin Hoshiiin, Maebashi, JPN; 2 Department of Hematology, National Hospital Organization Shibukawa Medical Center, Shibukawa, JPN

**Keywords:** adjuvant, paraffin plombage, reactive granuloma, foreign body reaction, igg4-related disease

## Abstract

We present a patient with IgG4-related disease (IgG4-RD) that developed after receiving extra-periosteal paraffin-embedded therapy for the treatment of pulmonary tuberculosis. The patient showed clinicopathological features consistent with IgG4-RD, including the enlargement of affected organs (salivary glands, lymph nodes, and retroperitoneal soft tissue mass), elevation of serum IgG4 levels, and infiltration of IgG4-positive plasma cells. The presence of reactive granulomas with foreign body giant cells (FBGCs) surrounding the paraffin-filled site suggested a type 2 helper T (Th2)-dominant immune response induced by the implanted biomaterial. Furthermore, paraffin, known to act as an adjuvant, may have played a role in activating the immune response and inducing IgG4-RD-like symptoms. This case highlights the potential relationship between foreign substances and the development of autoimmune diseases such as IgG4-RD.

## Introduction

IgG4-related disease (IgG4-RD) is an immune-mediated, chronic fibro-inflammatory disease characterized by the enlargement of the affected organs, elevation of serum IgG4, and significant infiltration of IgG4-positive plasma cells in the affected organs [1]. While the precise pathogenesis of IgG4-RD is not fully understood, type 2 helper T (Th2) is reportedly higher in proportion in IgG4-RD subjects [2,3], which may reflect their underlying allergic status. In addition, several candidate autoantigens have been identified, and multiple autoantigens may be involved in the pathogenesis [4]. We herein present a case of IgG4-RD that appeared decades after extra-periosteal paraffin plombage for pulmonary tuberculosis and discuss the potential pathophysiological connection between the two conditions.

## Case presentation

A 75-year-old male visited his family physician after having difficulty moving his left fingers for one month. He had undergone extra-periosteal paraffin plombage therapy 54 years earlier for pulmonary tuberculosis. Chest computed tomography (CT) revealed an enlarged plombage space with surrounding soft tissue density in his left chest cavity and bilateral axillary lymphadenopathy. He was referred to our hospital for concerns of tuberculous granuloma, sarcoma, or pyothorax-associated lymphoma.

**Figure 1:**
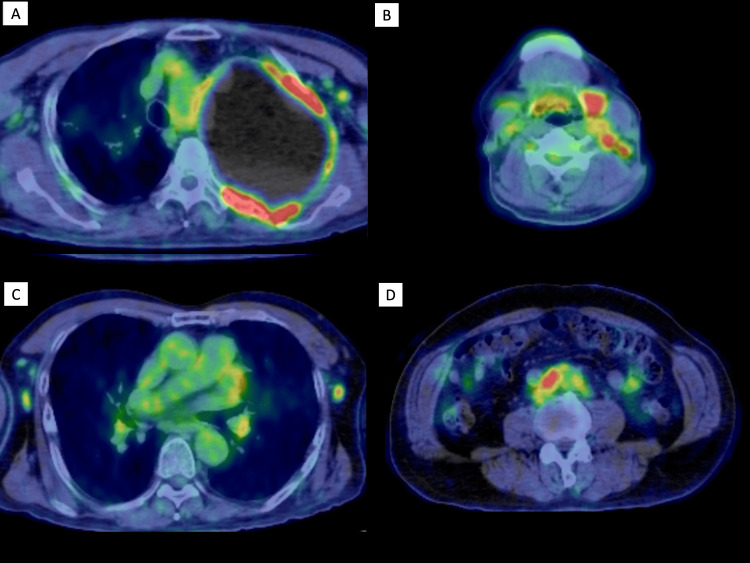
FDG PET-CT at admission. ^18^F-FDG PET-CT revealed the abnormal accumulation of FDG in a soft tissue mass around the plombage (A), salivary glands and cervical lymph nodes (B), axillary lymph nodes (C), and a retroperitoneal soft tissue mass around the abdominal aorta (D). Abbreviation: ^18^F-FDG PET-CT, ^18^F-fluorodeoxyglucose positron emission tomography-computed tomography.

Laboratory investigations are presented in Table 1. A peripheral blood analysis revealed a white blood cell count of 4,800/µL (neutrophils 53%, lymphocytes 28%, and eosinophils 10%), a normal hemoglobin level, and a normal platelet count. The patient’s liver and renal functions were normal; his serum C-reactive protein (CRP) level was 0.36 mg/dL. Tests for antinuclear antibody, anti-DNA antibody, antineutrophil cytoplasmic antibodies, and rheumatoid factor were all negative, and his complement component 3 (C3) and complement component 4 (C4) levels were within the normal limits. A tuberculosis T-cell spot (T-SPOT) assay was negative, whereas his soluble interleukin (IL)-2 receptor level was 1,065 U/mL. Whole-body ^18^F-fluorodeoxyglucose (FDG) positron emission tomography (PET)-CT showed the abnormal accumulation of FDG in the soft tissue mass around the plombage, enlarged salivary glands, cervical and axillary lymphadenopathy, and a retroperitoneal soft tissue mass surrounding the abdominal aorta (Figure 1).

**Table 1:** Laboratory investigations at admission. Abbreviations: WBC, white blood cell; RBC, red blood cell; TP, total protein; T-Bil, total bilirubin; AST, aspartate aminotransferase; ALT, alanine aminotransferase; LDH, lactate dehydrogenase; ALP, alkaline phosphatase; BUN, blood urea nitrogen; CRE, creatinine; CRP, C-reactive protein; MPO-ANCA, myeloperoxidase-antineutrophil cytoplasmic antibody; PR3-ANCA, proteinase-3-antineutrophil cytoplasmic antibody; C3, complement component 3; C4, complement component 4; CH50, 50% hemolytic complement; T-SPOT, T-cell spot of tuberculosis assay; SIL-2R, soluble IL-2 receptor.

Parameter	Result	Reference range
WBC (/μL)	4,800	3,900-9,800
Neutrophil (%)	53	30-78
Lymphocyte (%)	28	18-60
Monocyte (%)	9	3-10
Eosinophil (%)	10	0-5
Basophil (%)	1	0-1.2
RBC (×10^6^/μL)	4.44	4.27-5.20
Hemoglobin (g/dL)	15.2	13.5-17.6
Hematocrit (%)	44.1	39.8-51.8
Platelets (×10^4^/μL)	16.2	13.1-36.2
TP (g/dL)	8.0	6.7-8.3
T-Bil (mg/dL)	1.31	0.3-1.2
AST (U/L)	25	13-33
ALT (U/L)	14	8-42
LDH (U/L)	177	119-229
ALP (U/L)	69	115-359
BUN (mg/dL)	21.2	8-22
CRE (mg/dL)	0.88	0.6-1.1
Ca (mg/dL)	9.0	8.8-10.1
CRP (mg/dL)	0.36	0-0.14
Antinuclear antibody	<1:40	<1:40
Anti-DNA antibody (IU/mL)	<2	0-6
MPO-ANCA (U/mL)	<1	0-3.5
PR3-ANCA (U/mL)	<1	0-3.5
Rheumatoid factor (IU/mL)	10	0-15
C3 (mg/dL)	114	86-160
C4 (mg/dL)	20	17-45
CH50 (CH50/mL)	45.7	25-48
T-SPOT	Negative	Negative
SIL-2R (U/mL)	1,065	122-496
IgG (mg/dL)	3,209	861-1,747
IgA (mg/dL)	182	93-393
IgM (mg/dL)	48	33-183
IgG4 (mg/dL)	2,360	11-121
IL-6 (pg/mL)	2.5	0-4

**Figure 2:**
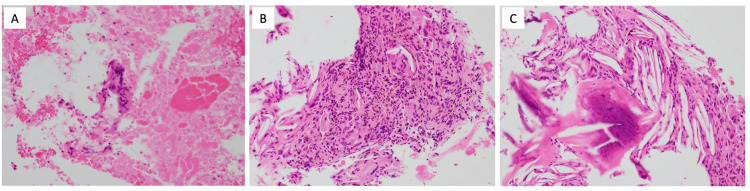
Biopsy specimens from the mass surrounding the site of paraffin plombage. Necrotic tissue and foreign body granuloma with the accumulation of lymphocytes, macrophages, and multinucleated giant cells (A-C: original magnification, ×200). There was no evidence of cancer or tuberculosis, and there were very few IgG4-positive plasma cells.

**Figure 3:**
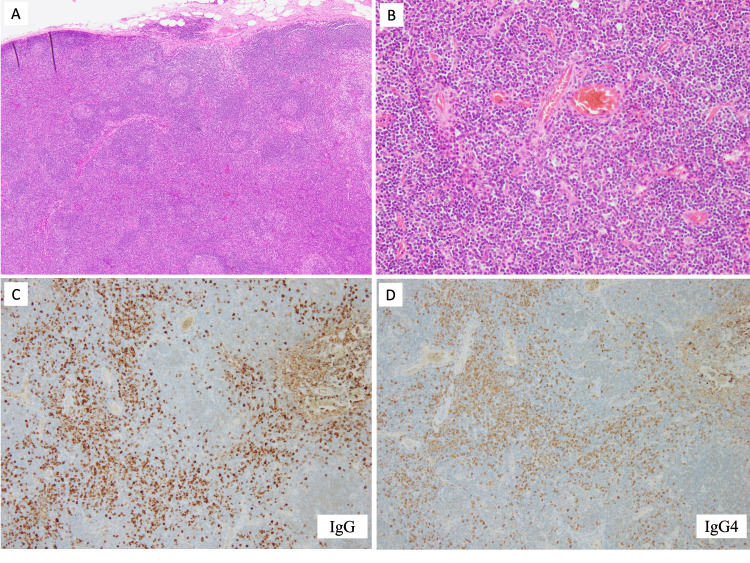
Biopsy specimens of the left axillary lymph node. Interfollicular expansion with normal to small germinal centers (A: original magnification, ×40). A mixed infiltrate of small lymphocytes, immunoblasts, immature plasma cells, mature plasma cells, and scattered eosinophils was observed in the interfollicular zone (B: original magnification, ×100). Immunohistochemistry showed a high IgG4 /IgG ratio (C, D: original magnification, ×100).

We then performed a left axillary lymph node biopsy and needle biopsy on the mass surrounding the plombage. Reactive granuloma was diagnosed based on the examination of biopsy specimens from the mass surrounding the plombage, indicating a foreign body reaction to paraffin leaking from the plombage (Figure 2). There were no indications of malignancy or tuberculosis. However, the left axillary lymph node biopsy revealed a significant number of infiltrating lymphocytes and IgG4-positive plasma cells (IgG4/IgG ratio 80%) (Figure 3), leading to a diagnosis of IgG4-related lymphadenopathy (type III: interfollicular expansion and immunoblastic type) [5]. Laboratory tests also showed elevated serum IgG and IgG4 levels (3,209 and 2,360 mg/dL, respectively) but a normal level of IL-6. According to the 2020 revised comprehensive diagnostic criteria for IgG4-RD [6], he was diagnosed with “definite” IgG4-RD.

Due to concerns about his advanced age and general condition, we decided against removing the plombage. Meanwhile, to treat IgG4-RD, oral prednisolone was started at a dose of 0.5 mg/kg/day and then tapered off at a rate of 5 mg every two weeks. His serum IgG4 levels gradually declined and normalized after two months. On follow-up ^18^F-FDG PET-CT four months later, the salivary glands, cervical and axillary lymph nodes, and retroperitoneal soft tissue mass showed less FDG accumulation; however, the amount of FDG in the soft tissues surrounding the paraffin plombage remained unchanged.

## Discussion

In the 1940s and 1950s, surgical treatment of pulmonary tuberculosis, particularly collapse therapies, such as thoracoplasty and extra-periosteal plombage, was widely used around the world [7]. Nearly 30 different materials, including paraffin wax, polythene bags, and methyl-methacrylate (Lucite) balls, were reportedly used to keep the affected lungs collapsed, and some patients still have these fillings inside their bodies [8]. The materials used in plombage can leak or rupture or cause infection over time, leading to late complications such as pyothorax, hemothorax, and subcutaneous masses brought on by the migration of foreign bodies [9].

Our case also had extensive reactive granulomas containing foreign body giant cells (FBGCs) surrounding the site of paraffin plombage. A foreign body granuloma is a Th2-predominant immune response triggered by implanted biomaterials in the body [10]. Briefly, mast cells and Th2 cells first produce inflammatory cytokines (IL-4 and IL-13) at the transplant site, which induce macrophage fusion and the formation of FBGCs. Such fused FBGCs subsequently produce fibrosis-promoting factors (transforming growth factor-beta (TGF-β) and platelet-derived growth factor (PDGF)) and anti-inflammatory cytokines (IL-10 and IL-1ra), which help to reduce inflammation and encapsulate foreign substances.

In addition, paraffin is known to act as an adjuvant, which is a substance that boosts antigen-specific immune responses and triggers a characteristic inflammatory cascade in vivo [11]. To our knowledge, there are no reports of IgG4-RD being proven as an apparent human adjuvant disease in the literature. However, several case reports and cohort studies have focused on the relationship between IgG4-RD and chronic occupational antigen exposure, such as silicosis and asbestos-related pleural disease [12-16]. Silicosis patients frequently have autoimmune diseases, such as rheumatoid arthritis, systemic lupus erythematosus, and scleroderma, which have been partially explained as adjuvant-type effects of silica [17]. Similarly, asbestos nanoparticles could trigger a Th2 immune response, such as mast cell activation, by acting as a Th2 adjuvant [18]. The Th2 immune response is important in IgG4-RD, implying a possible adjuvant-mediated mechanism linking IgG4-RD and asbestos-related pleural disease. Furthermore, the production of IgG4 is regarded as one of the anti-inflammatory tolerance mechanisms that can be activated following prolonged or intense antigen exposure [19]. Based on these findings, we presumed that paraffin served as an adjuvant in this case, activating the immune response and causing systemic symptoms similar to IgG4-RD. The patient was incapable of removing the paraffin due to his deteriorated general condition. The efficacy of foreign body removal in ameliorating human adjuvant disease remains controversial. Conversely, in a case of IgG4-RD after silicon injection into the temporomandibular joint, a treatment regimen consisting of prednisolone followed by rituximab was reported to have reduced the tumor [20].

## Conclusions

The etiology of IgG4-RD remains to be elucidated, and various factors, including genetic predisposition and environmental triggers, may contribute to the pathogenesis of this disease. The clinicopathological alterations consistent with IgG4-RD observed in this case might have been influenced by a persistent immune response to the implanted paraffin. Despite significant changes in the treatment of pulmonary tuberculosis over the past 50 years, paraffin plombage in the body may have remained a foreign substance and continued to chronically stimulate the systemic immune system. Our case may provide insight into the autoimmune developmental process of IgG4-RD triggered by foreign substances.
